# Difference of uveal parameters between the acute primary angle closure eyes and the fellow eyes

**DOI:** 10.1038/s41433-018-0056-9

**Published:** 2018-03-01

**Authors:** Xingyi Li, Wei Wang, Wenbin Huang, Shida Chen, Jiawei Wang, Zhonghao Wang, Yaoming Liu, Mingguang He, Xiulan Zhang

**Affiliations:** State Key Laboratory of Ophthalmology, Zhongshan Ophthalmic Center, Sun Yat-Sen University, Guangzhou, 510060 China

## Abstract

**Purpose:**

To measure the anterior and posterior ocular biometric characteristics concurrently and to explore the relationship between iris, ciliary body and choroid in acute primary angle closure eyes (APAC) and fellow eyes.

**Methods:**

It is a prospective, cross-sectional study. Thirty patients with recent APAC were finally enroled in it. Anterior and posterior uveal parameters were measured simultaneously by anterior segment optical coherence tomography (AS-OCT), swept-source optical coherence tomography (SS-OCT) and ultrasound biomicroscopy (UBM). The parameters were measured including: pupil diameter (PD); iris thickness, curvature (ICURV), area (IAREA); anterior chamber depth (ACD), width (ACW), area (ACA), volume (ACV); lens vault (LV); choroidal thickness and retinal thickness; maximum ciliary body thickness (CBTmax); ciliary body thickness at the point of the scleral spur (CBT0) and 1000 mm away (CBT1000); anterior placement of the ciliary body (APCB); and trabecular-ciliary angle (TCA).

**Results:**

Compared with fellow eyes, APAC eyes had narrower anterior biometric parameters and presented with smaller anterior segment parameters (including ACD and ACW); (*p* < 0.01)), smaller IAREA and ICURV (*p* < 0.001), larger LV (*p* = 0.035), thinner ciliary body and less anterior ciliary process (*p* < 0.01). After adjustment for potential confounders (axial length, spherical equivalent and PD), APCB was positive correlated with choroidal thickness and CBT, and CBT was positive correlated with choroidal thickness.

**Conclusions:**

Compared with fellow eyes, APAC eyes had narrower anterior biometric parameters, thinner ciliary body and smaller iris area and curvature. APCB, CBT and choroidal thickness were positively correlated. However, further studies are required before these conclusions are generalised.

## Introduction

Acute primary angle closure (APAC) is characterised by a sudden elevation in intraocular pressure (IOP) accompanied by other findings, such as corneal oedema, a shallow anterior chamber and typical symptoms, such as blurred vision, severe ocular pain or headache, nausea and vomiting. It is considered an abnormal anatomic disorder [1]. Previous studies have used anterior segment optical coherence tomography (AS-OCT) [2] to evaluate the various anterior segment parameters in angle closure eyes. Pupillary block and angle crowding were proposed as the two main mechanisms underlying the pathogenesis of angle-closure glaucoma, although other structures, such as the lens, iris and ciliary body, have been shown to contribute to the presence of angle closure.

Recent studies have suggested that the choroid may play an important role in APAC [3, 4]. Swept-source optical coherence tomography (SS-OCT) is a relatively new innovative technology that is used to visualise and quantify choroidal thickness [5, 6]. However, until recently, no study has attempted concurrent measurement of the anterior and posterior parts of the uvea (iris, ciliary body and choroid) or an evaluation of the relationship between them. The aim of this study was to investigate the possible associations among the different parts of the uvea in APAC eyes, focusing on the differences in the iris, ciliary body and choroid between APAC and fellow eyes.

## Methods

### Subject recruitment

This was a prospective, cross-sectional study performed in the Zhongshan Ophthalmic Centre of Sun Yat-sen University (Guangzhou, China) and approved by the institutional review board. The study was complied with the Declaration of Helsinki and all participants provided informed written consent.

Patients suffering unilateral APAC attacks were recruited to participate in the study. APAC was defined based on previous reported criteria: (1) the presence of at least two of the following symptoms: ocular pain, periocular pain or headache; (2) nausea and/or vomiting; (3) a previous history of intermittent blurred vision or halos around lights; (4) raised IOP of at least 25 mmHg; (5) and the presence of at least three of the following signs [7]: conjunctival injection, corneal epithelial oedema, mid-dilated unreactive pupil and/or a shallow anterior chamber. A primary angle closure suspect (PACS) eye was defined as a pigmented trabecular meshwork in the eye not visible or iridotrabecular contact (ITC) of 180 degrees or more under non-compressive gonioscopy (Goldmann), with normal IOP (<21 mmHg) and without peripheral anterior synechiae or glaucomatous neuropathy [2, 8, 9]. The choroidal thickness of all APAC eyes and their fellow PACS eyes were measured using SS-OCT. All imaging examinations were performed in 24 h right after the reduction of IOP by medication treatment (anti-inflammatory drops and/or a systemic steroid and hypertonic drugs) in affected eyes in order to ensure the corneal transparency, but before any procedures of peripheral iridectomy, iridoplasty or trabeculectomy. However, we excluded patients with a current use of topical or systemic medications that could affect the angle or the pupillary reflex, such as using topical pilocarpine in recent 5 days [10]. Recruitment was restricted to patients whose opacity in the APAC eyes became clear after preliminary treatment, and whose fellow eyes were diagnosed with PACS; recruitment was prospective and consecutive, from January 2016 to August 2016.

Patients with any of the following conditions were excluded: a secondary angle closure because of lens disorder or tumour; a history of intraocular surgery or trauma; high myopia with a spherical equivalent (SE) (>−6 dioptres [D]) or hyperopia (>+ 3 dioptres [D]); any retinal or choroidal disease; opacities of the optical media; systemic disease, such as diabetes or hypertension; inability to tolerate examinations.

### Examinations

All subjects underwent detailed ocular examinations, including best-corrected visual acuity, a slit-lamp examination, a stereoscopic optic disc examination with a 90-diopter lens, and IOP measurement using Goldmann applanation tonometry. Gonioscopy was performed in the dark using a Goldmann 1-mirror lens at high magnification by one examiner (XZ). They also underwent refractive error examination using an auto refractometer (KR-8900 version 1.07; Topcon Corporation, Tokyo, Japan) and axial length measurements using partial optical coherence interferometry (IOL-Master; Carl Zeiss Meditec, La Jolla, CA, USA).

We performed all examinations following standard operation procedures, and AS-OCT, SS-OCT and UBM were conducted on the same morning around 10 am. We performed the AS-OCT and SS-OCT, then the contact examinations, such as gonioscopy and UBM last. The duration of all examinations was ~40 min.

### AS-OCT imaging and measurements

**Fig. 1:**
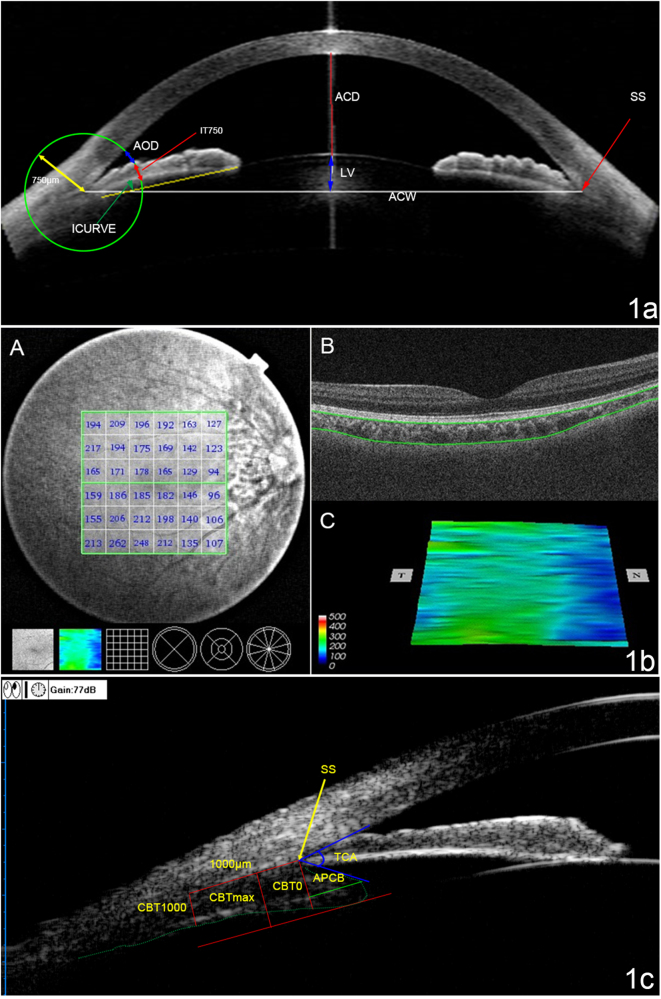
**a** AS-OCT image showing the automatic measurements of anterior chamber depth (ACD), angle opening distance at 750 μm from the scleral spur (AOD750), iris thickness at 750 μm from the scleral spur (IT750), iris curvature (ICURV), anterior chamber width (ACW), and lens vault (LV). **b** SS-OCT image showing the measurements of choroidal thickness. **A** A choroidal thickness map of the 6 × 6 mm area centred on the fovea was created. The mean choroidal thickness was obtained for each sector. **B** Automatic placement of the chorioscleral border made using automatic built-in software in one of the B-scan images of the 3D data set. **C** Choroidal topographic map of the 6 × 6 mm area. **c** Determination of ciliary body parameters on ultrasound bio-microscopy. The figure showed the measurement of maximum ciliary body thickness (CBTmax), ciliary body thickness at the point of the scleral spur (CBT0), ciliary body thickness at a distance of 1000 mm (CBT1000) from the scleral spur, anterior placement of the ciliary body (APCB) and trabecular-ciliary angle (TCA)

AS-OCT imaging and image quantifications were conducted as described in our previous studies [2, 9, 11]. AS-OCT (Visante OCT; Carl Zeiss Meditec, Dublin, CA, USA) was measured under dark room conditions (0 lux) by a single-experienced operator who was masked to the diagnosis of the patients. The standard anterior segment single-scan protocol was performed, and the images were centred on the pupil to obtain a single-cross-sectional horizontal scan (nasal-temporal angles at 0°–180°). During the examination, the examiner adjusted the noise and saturation and optimised the polarisation to obtain the best quality image. Several scans were obtained for each subject in order to choose the image with the fewest artefacts. The images were then processed using the Zhongshan Angle Assessment Program (ZAAP, Guangzhou, China) [2]. The only operation performed on each image was to determine the location of the two scleral spurs. The following parameters, shown in Fig. 1a, were derived automatically from the software: pupil diameter (PD), iris thickness at 750 μm from the scleral spur (IT750), iris curvature (ICURV), iris area (IAREA), ACD, ACW, ACA, ACV and lens vault (LV). The image analyst repeated the operation on one image three times to acquire the average data.

### SS-OCT measurements

The images of the macular region were obtained using an SS-OCT instrument (DRI OCT-1; Topcon, Tokyo, Japan) just after the AS-OCT examination. The SS-OCT system uses a tuneable laser as a 1050 nm light source with a 100 nm tuning range and had an 8-μm axial resolution in tissue. More details about the device have been described elsewhere [11, 12].

A three-dimensional (3D) imaging scan protocol was used to measure the macular choroidal thickness. The 3D imaging data set was acquired with a 6 × 6-mm raster scan centred on the fovea and composed of 256 B-scans, each consisting of 256 A-scans (a total of 65,536 axial scans/volume). The scan protocol was repeated three consecutive times on the same eye. The patient and device were repositioned after each scan. Measurements of both eyes of each patient were obtained through undilated pupils. Only images having a quality score of 45 (of 160) or more were included in the analysis. The effects of diurnal variations were reduced by performing all examinations in the morning at ~10 am [13]. Image artefacts, such as motion artefacts, signal loss resulting from blinking and segmentation failure, were excluded, and the number of scans with an artefact and the type of the artefact were recorded [5].

Choroidal and retinal thickness measurements were performed using built-in software (9.12.003.04). A 6 × 6 mm thickness map of the choroid was created using automated segmentation. The 6 × 6 grid was used for the thickness map (Fig. 1b), and the mean regional thicknesses of the choroid were calculated for the 36 sectors of the grid.

### Ultrasound biomicroscopy

The UBM (model SW-3200L; Tianjin Suowei Electronic Technology Co, Ltd., Tianjin, China) used in this study was equipped with a single-element mechanical linear scanner, as described in our previous study about the ciliary body. UBM examinations and measurements were performed on the same morning after the AS-OCT and SS-OCT examinations, by one experienced physician (Z.W.) who was masked to the clinical diagnosis. We performed the UBM examinations with the patients lying in a supine position in a dimly lit room (illumination 60–70 lux). Radial scans at the 12, 3, 6 and 9 o’clock positions centred over the limbus and perpendicular sulcus-to-sulcus scans over the pupil centre were obtained. Several scans were obtained to choose the clearest image. The following data were measured at every clock position: (1) maximum ciliary body thickness (CBTmax), which was defined as the distance from the most inner point of the ciliary body to the inner wall of the sclera or its extended line; (2) ciliary body thickness at the point of the scleral spur (CBT0) and at a distance of 1000 mm (CBT1000) from the scleral spur; (3) anterior placement of the ciliary body (APCB), which was the distance from the most anterior point of the ciliary body to the vertical line from the inner wall of the sclera through the scleral spur; and (4) trabecular-ciliary angle (TCA), which was the angle between the posterior corneal surface and the anterior surface of the ciliary body, as described previously [14] (Fig. 1c). Every parameter was measured three times, and the median values were recorded. The results of every parameter were the averages of the parameter at the 4 o’clock position. The accuracy and repeatability of the ciliary body measurements of the UBM have been confirmed previously [14].

### Statistical analysis

The minimum required sample size for the study was calculated based on the previous study comparing the anterior chamber and choroidal thickness parameters of APAC and fellow eyes. Considering the previous study results for macular choroidal thickness, 21 pairs of eyes would be needed to detect a 5% change in choroidal thickness with the power of a 95% confidence level [3]. Based on the research on anterior segment parameters determined using AS-OCT, 30 pairs of eyes would be needed to detect a 5% change in ACD with the power of a 95% confidence level [15].

Statistical analyses were performed using SPSS software version 17.0 (SPSS, Inc., Chicago, IL, USA). The means and standard deviations of the above parameters were calculated. The variance between the APAC eyes and their fellow eyes are similar, and paired *t*-tests were used to detect the differences between the two groups. Univariate and multivariate linear regression was used to determine the relationship between the biometric measurements of the iris, the choroid and the parameters of ciliary body measurements separately in APAC eyes and fellow eyes. A *p*-value of <0.05 was considered significant.

## Results

In total, 34 patients with unilateral APAC were initially enroled. Four patients were excluded because a clear SS-OCT image or the UBM imagescould not be obtained due to a lack-of-clarity in the optical media and poor patient compliance. Ultimately, 30 Chinese patients (30 pairs of eyes) with unilateral APAC and fellow eyes defined as PACS eyes were analyzed.

The demographic data are presented in Table 1. The baseline data between APAC and fellow eyes are in Table 2. The mean SE was +1.6 D (SD ± 1.7 D) in the APAC eyes and +1.8 (SD ± 1.5 D) in the PACS eyes. The mean axial lengths (AL) of the APAC and PACS eyes were 22.1 mm (SD ± 0.8 mm) and 22.1 mm (SD ± 0.9 mm), respectively. No significant differences were found for any of the variables between the two groups, as summarised in Table 2.

**Table 1:** Demographic and baseline characteristics of patients

Characteristic	Subjects of study
*N* patients (*N* eyes)	30 (30)
Mean age, y (SD)	60.8 ± 7.8
Sex, male/female	5/25
Laterality of affected eye, right/left	12/18
Duration of experienced attack, d (SD)	8.9 ± 6.0
Presenting IOP, mmHg, affected eye (SD)	42.3 ± 6.3
IOP at imaging, mmHg, affected eye (SD)	13.6 ± 4.5
IOP at imaging, mmHg, fellow eye (SD)	12.2 ± 3.5

*IOP* intraocular pressure, *SD* standard deviation

**Table 2:** Comparing the baseline data and biometric measurements of APAC eyes and fellow eyes

	APAC eye		Fellow eye		
	Mean	SD	Mean	SD	*P*-value
IOP at imaging (mmHg)	13.6	4.5	12.2	3.5	0.086
Spherical equivalent (D)	1.6	1.7	1.8	1.5	0.620
PD (mm)	4.6	1.6	4.3	1.5	0.402
AL (mm)	22.1	0.8	22.1	0.9	0.669
CBTmax (mm)	0.87	0.14	0.93	0.11	0.002**
CBT0 (mm)	0.84	0.13	0.90	0.10	0.004**
CBT1000 (mm)	0.58	0.10	0.62	0.09	0.066
APCB (mm)	0.68	0.21	0.75	0.16	0.021*
TCA (degrees)	47.97	9.74	48.46	7.09	0.782
IT750 (mm)	0.49	0.08	0.52	0.09	0.161
IT2000 (mm)	0.46	0.12	0.49	0.07	0.207
IAREA (mm^2^)	1.37	0.29	1.68	0.32	<0.001**
ICURV (mm)	0.22	0.15	0.36	0.17	0.001**
ACD (mm)	1.74	0.21	1.84	0.19	<0.001**
ACW (mm)	11.02	0.44	11.17	0.44	0.005**
ACA (mm^2^)	12.17	1.84	12.83	3.30	0.216
ACV (mm^3^)	73.73	14.69	75.66	19.08	0.483
LV (μm)	1092.7	174.5	1030.6	164.3	0.035*
CT (μm)	234.01	84.30	221.02	70.40	0.408
Retina (μm)	267.90	20.64	267.43	10.04	0.895

Data are expressed as the mean ± SD

*IOP* intraocular pressure, *D* diopter, *PD* pupil diameter, *AL* axial length, *CBTmax* maximum ciliary body thickness, *CBT0* ciliary body thickness at the point of the scleral spur, *CBT1000* ciliary body thickness at 1000 μm from the scleral spur, *APCB* anterior placement of the ciliary body, *TCA* the trabecular-ciliary process angle, *IT750* iris thickness at 750 μm from the scleral spur, *IT2000* iris thickness at 2000 μm from the scleral spur, *IAREA* iris area, *ICURV* iris curvature, *ACD* anterior chamber depth, *ACW* anterior chamber width, *ACA* anterior chamber area, *ACV* anterior chamber volume, *LV* lens vault, *CT* choroidal thickness, *SD* standard deviation, *95% CI* 95% confidence interval

* Paired *t*-test. *P* < 0.05

** *p* < 0.01

### Iris and choroid parameters

The parameters measured using AS-OCT and SS-OCT are shown in Table 2. Compared with the fellow eyes, the APAC eyes had a smaller IARE (*p* < 0.001), ICURVE (*p* = 0.001), ACD (*p* < 0.001) and ACW (*p* = 0.005) and a larger LV (*p* = 0.035). No difference was noted in choroidal thickness and retina thickness at the macular region between the APAC eyes and the fellow eyes.

### Ciliary body parameters measured using UBM

As shown in Table 2, the APAC eyes had thinner ciliary body thickness (CBTmax, *p* = 0.002; CBT0, *p* = 0.004) compared with the fellow eyes and less anterior ciliary body processes (APCB, *p* = 0.021). No difference was noted for the TCA between the APAC eyes and the fellow eyes.

### Associations between choroidal thickness, iris parameters, and ciliary body parameters

Further analyses were performed for the anterior segment anatomic parameters (LV, PD, IT750, IAREA and ICURV) and ciliary body parameters (CBTmax, APCB and TCA) associated with the choroidal thickness of the APAC eyes (Table 3) and of the fellow eyes (Table 4). Only APCB was identified positive correlated with choroidal thickness, after adjusting for AL, SE and PD (*p* < 0.05), suggesting that the eyes with more anterior ciliary body have thicker choroid in both APAC eyes and fellow eyes.

**Table 3:** Univariate and multivariate linear regression for associated between choroidal thickness and iris and ciliary body parameters of APAC eyes

	Univariate		Multivariate	
	β^a^ (95% CI)	*P*-value^a^	β^b^ (95% CI)	*P*-value^b^
*CT*				
SE	13.11 [−5.44, 31.66]	0.159	—	—
IOP	−0.59 [−7.48, 6.31]	0.860	—	—
AL	−33.23 [−69.98, 3.53]	0.075	—	—
PD	0.82 [−19.16, 20.79]	0.934	—	—
CBTmax	168.05 [−57.77, 393.87]	0.139	—	—
APCB	154.32 [12.40, 296.25]	0.034^a^	148.57 [2.74, 294.41]	0.046^b^
IT750	250.38 [−122.81, 623.57]	0.063	—	—
IARE	54.08 [−57.08, 165.23]	0.328	—	—
ICURVE	23.12 [−193.95, 240.18]	0.829	—	—
LV	0.071 [−0.11, 0.26]	0.440	—	—
ACD	−33.10 [−191.04, 124.85]	0.671	—	—
*CBTmax*				
SE	0.00 [−0.03, 0.03]	0.906	—	—
IOP	−0.01 [−0.02, 0.00]	0.202	—	—
AL	0.01 [−0.06, 0.07]	0.804	—	—
PD	−0.01 [−0.04, 0.03]	0.618	—	—
APCB	0.34 [0.12, 0.56]	0.003^a^	0.36 [0.12, 0.60]	0.005^b^
IT750	0.23 [−0.40, 0.86]	0.465	—	—
IARE	0.14 [−0.04, 0.31]	0.132	—	—
ICURVE	0.26 [−0.09, 0.60]	0.136	—	—
LV	0.00 [0.00, 0.00]	0.993	—	—
ACD	0.19 [−0.07, 0.44]	0.143	—	—

Multivariate linear regression have adjusted for SE, AL and PD

Insignificant variables were not present in multivariate regressions

^a^ Univariate linear regression *p* < 0.05

^b^ Multivariate linear regression *p* < 0.05

**Table 4:** Univariate and multivariate linear regression for associated between choroidal thickness and iris and ciliary body parameters of fellow eyes

	Univariate		Multivariate	
	β^a^ (95% CI)	*P*-value^a^	β^b^ (95% CI)	*P-*value^b^
*CT*				
SE	−11.64 [−29.34, 6.07]	0.189	−17.73 [−34.13, −1.33]	0.035^b^
IOP	5.13 [−2.45, 12.70]	0.176	—	—
AL	−20.69 [−50.35, 8.97]	0.164	−31.56 [−58.81, −1.33]	0.025^b^
PD	1.08 [−16.52, 18.68]	0.126	—	—
CBTmax	197.35 [−33.46, 428.16]	0.091	—	—
APCB	182.88 [25.76, 340.00]	0.024^a^	200.32 [54.35, 346.29]	0.009^b^
IT750	−74.25 [−370.28, 221.78]	0.611	—	—
IARE	21.58 [−63.97, 107.12]	0.609	—	—
ICURVE	105.79 [−53.14, 264.71]	0.184	—	—
LV	0.05 [−0.12, 0.21]	0.557	—	—
ACD	−64.25 [−204.87, 76.38]	0.357	—	—
*CBTmax*				
SE	−0.01 [−0.04, 0.02]	0.623	−0.01 [−0.04, 0.02]	0.493
IOP	−0.01 [−0.02, 0.01]	0.273	—	—
AL	−0.02 [−0.07, 0.03]	0.388	−0.03 [−0.08, 0.02]	0.281
PD	0.01[−0.02, 0.03]	0.715	0.01 [−0.02, 0.04]	0.700
APCB	0.10 [−0.18, 0.37]	0.474	—	—
IT750	0.17 [−0.30, 0.64]	0.470	—	—
IARE	0.03 [−0.11, 0.16]	0.699	—	—
ICURVE	−0.00 [−0.26, 0.26]	0.997	—	—
LV	0.00 [0.00, 0.00]	0.690	—	—
ACD	0.19 [−0.02, 0.41]	0.076	—	—

Multivariate linear regression have adjusted for SE, AL and PD

Insignificant variables were not present in multivariate regressions

^a^ Univariate linear regression *p* < 0.05

^b^ Multivariate linear regression *p* < 0.05

### Associations between ciliary body parameters and anterior segment anatomic parameters

The regression analyses, after adjustment for potential confounders of AL, SE and PD, showed that APCB was positive correlated with ciliary body thickness in APAC eyes (*p* < 0.001). However, this finding was not evident in the fellow eye group. Therefore, ciliary bodies that are more anterior are thicker, especially in APAC eyes.

## Discussion

APAC is an acute attack of angle closure that dramatically increases IOP. Anatomical changes in the iris and lens of APAC eyes have been previously reported. To the best of our knowledge, this is the first study to provide a simultaneous quantification of both anterior chamber angle structures and uveal parameters, including the iris and the ciliary body, and choroidal thickness in APAC eyes and fellow eyes. Our observations of shallower ACD, thicker lens, high LV and smaller IAREA of APAC eyes compared to fellow eyes are corroborated with previous AS-OCT studies [2, 15, 16].

Although, AS-OCT is a simple, non-contact alternative for the imaging of the anterior chamber angle, iris pigment epithelium limits light transmission and thus may limit the visualisation of structures posterior to the iris. UBM is able to reveal the characteristics of some anterior chamber angle structures posterior to the iris. The previous studies using UBM showed that a thick iris and ciliary body have a higher odds ratio of having angle closure when compared to normal controls [17, 18]. However, the information of differences in ciliary body parameters between APAC eyes and fellow eyes is limited.

Our study showed that APAC eyes had thinner ciliary bodies and smaller anterior ciliary processes than in the fellow eyes.. He et al. [9] demonstrated that Chinese persons have thinner ciliary bodies than American Caucasians and postulated that this may be associated with the higher prevalence of angle closure in the Chinese population. In addition, a quantitative study of the ciliary body in eyes with malignant glaucoma showed thinner ciliary body in eyes with malignant glaucoma and in their fellow eyes, which might indicate a predisposing factor for malignant glaucoma [14]. And Wang et al. [19] found that ciliary bodies were thinner and more anteriorly rotated in eyes with APAC, as well as in their fellow eyes, which was consistent with our results. At the same time, we found that APAC eyes had smaller anterior ciliary processes. The CBT0 of the APAC eyes was significantly thinner than that of the fellow eyes, but no difference was noted between the APAC and fellow eyes in terms of CBT1000, which meant that the ciliary body was thinner just near the scleral spur. The extension of the ciliary body might cause the ciliary process to recede. We speculated that a thin ciliary body may cause a loosening of zonules and a more variable position of the lens [9]. However, whether a thinner ciliary body is the cause or a result of APAC requires further longitudinal study.

Our previous research demonstrated an obviously greater choroidal thickness in APAC eyes than in fellow eyes, and greater choroidal thickness in PAC eyes than normal controls, which supported the hypothesis of choroid thicken in angle closure [3, 4, 20, 21]. However, in this study, we only found a slightly greater choroidal thickness in APAC eyes, although with no statistical difference. Several reasons could explain the inconsistencies between our presenting findings and those of the previous study. In this study, we measured choroidal thickness after the IOP of the APAC eyes became normal (below 21 mmHg) to make sure the cornea transparency, which took a much longer time after the APAC attack (8.9 ± 6.0 days) than in the previous study (6.5 ± 2.4 days), and we possibly lost the time point of the most thickened choroid. According to the IOP changes, it might have some dramatic changes of the ocular blood flow to the choroid, which caused the change of the choroidal thickness. In previous studied, the choroidal flow measured by laser Doppler flowmetry decreased when the IOP increased in rabbits [22]. Hata et al. [23] proved that the IOP increase may have caused choroidal hypoperfusion in primary angle closure glaucoma eyes. Song et al. [24] had the same finding. We could speculate that the choroidal thickness before APAC attack of the affected eyes was much greater, and the difference from fellow eyes would be more significant. The IOP raised when APAC attack, which made the uveal blood flow reduced and the choroid thinned. However, we need further longitudinal study about the blood flow change shortly after APAC attack.

Uveal effusion might be another potential source of the increased choroidal thickness in APAC eyes. Kumar et al.[25] found that 25% of Asian eyes with APAC presented with uveal effusion. However, it is unknown if uveal effusion is a cause or effect of APAC. In our study, we found no sign of uveal effusion in either the APAC or fellow eyes, and all of the APAC eyes received anti-inflammatory drops and/or a systemic steroid to control the inflammation, which potentially may have reduced the uveal effusion.

We know that the iris, the ciliary body and the choroid are components of the uvea, which is the vascular middle layer of the eye. In our study, we measured the parameters of the uvea, including the iris, the ciliary body and the choroid, in order to research the correlation of different parts of the uvea during an APAC attack. We hypothesised that the blood supply to these tissues would dynamically change and cause some anatomical changes. However, after adjusting for potential influencing factors (including SE, AL and PD), no association was found between the macular choroidal thickness and iris parameters, and the same between the ciliary body and iris parameters, which might be a result of insufficient statistical power.

In addition, the correlation between the ciliary body and the choroid was subtle. APCB was identified positive correlated with choroidal thickness, which could be explained by the choroidal expansion hypothesis: the choroid would expand and push the ciliary body forward. We can speculate that the expansion of the choroid affected the ciliary body size and location. Based on the weakness zonules and a variable position of the lens that was mentioned earlier, lens in some eyes would move much anterior, and make the anterior chamber more narrow, even closed. However, further longitudinal studies are needed to evaluate the dynamic changes, especially in primary angle closure patients.

At the same time, APCB was positive correlated with CBTmax, which might be easily understood as a large ciliary body accompanying with an anterior ciliary process. According to the current findings, we speculated that the thickness of anterior choroid near the pars plana of ciliary body might play more role in affect the anterior chamber changes and angle closure. Therefore, further studies focus on anterior choroid changes would be designed.

Some potential limitations in our study should be mentioned. First, the sample size was limited; however, most parameters of the anterior chamber and the iris were sufficient to show a significant difference between the APAC eyes and the fellow eyes, which is consistent with previous studies. Second, this cross-sectional study made it difficult to establish temporal or causal relationships. Therefore, a prospective longitudinal study is needed to address the cause-and-effect relationship between the dynamic changes of different parts of the uvea. Third, the average duration of attacks experienced by the patients was longer than in our previous study [3] and the SD of the average duration was large. However, in order to reduce the probable influence factor, we have divided the patients into current APAC groups (duration <7 days) and previous APAC groups (duration >7 days), and then separately calculated the differences between the APAC eyes and their fellow eyes; this result still agreed with the result calculated totally (data not shown). Finally, the limitations of UBM measurements were obvious. Systemic errors in ultrasound digital image distance measurements, including inherent image pixelation errors and modality-specific registration errors, were unavoidable [26]. Therefore, in our study, the same settings (including measuring instrument, illumination and scanning protocol) were used in every case. All scans and measurements were performed by the same experienced physician (Z.W.). The intraobserver repeatability and reproducibility have been validated [14].

## Conclusion

In summary, this study is the first to use AS-OCT, SS-OCT and UBM for the concurrent measurement of anterior and posterior biometric parameters of uvea. Compared with fellow eyes, APAC eyes had narrower anterior biometric parameters, and a thinner ciliary body, a smaller IAREA area and a smaller ICURVE. APCB was positive correlated with choroidal thickness and CBT. However, no correlation was noted between other parameters of different parts of the uvea. The choroidal thickness might dramatically change during APAC attack. Our attempt was to find some relationship between the biometric features of the iris, the ciliary body and the choroid, and the results indicated that changes in the different parts of the uvea might be associated with APAC attacks. However, this needs to be investigated in future studies before a generalisable conclusion can be drawn.

## Summary

### What was known before

- Compared with fellow eyes, APAC eyes had narrower anterior biometric parameters.

### What this study adds

- Compared with fellow eyes, APAC eyes had thinner ciliary bodies, and smaller iris areas and iris curves.
- APCB, CBT and choroidal thickness were positively correlated. No significant association between other parameters in different parts of the uvea was detected.
